# Diagnostic accuracy of *Mycobacterium tuberculosis* antigen-induced interferon-γ-inducible protein 10 for latent tuberculosis infection among high-risk groups: a systematic review and meta-analysis

**DOI:** 10.3389/fimmu.2026.1951983

**Published:** 2026-09-28

**Authors:** Lan Huang, Yong Hu, Qian Mao, Long Ge, Xia Qiu

**Affiliations:** 1 Department of Pediatrics, West China Second University Hospital, Sichuan University, Chengdu, China; 2 Key Laboratory of Birth Defects and Related Diseases of Women and Children, Sichuan University, Ministry of Education, Chengdu, China; 3 Department of Social Medicine and Health Management, School of Public Health, Lanzhou University, Lanzhou, China

**Keywords:** latent tuberculosis infection, high-risk groups, interferon-γ-inducible protein 10, *Mycobacterium tuberculosis* antigen-induced, diagnostic accuracy

## Abstract

**Background:**

Latent tuberculosis infection (LTBI) affects nearly a quarter of the world’s population, yet current diagnostic tools are suboptimal. Interferon-γ-inducible protein 10 (IP-10) has been proposed as a candidate biomarker, but its diagnostic value for LTBI among high-risk groups is unclear. To address this, we performed a systematic review and meta-analysis to assess the accuracy of *Mycobacterium tuberculosis* (Mtb) antigen-induced IP-10 for detecting LTBI among high-risk groups.

**Methods:**

PubMed, Web of Science, Embase, Scopus, and the Cochrane Library were searched up to April 2026. Cohort and cross-sectional studies evaluating Mtb antigen-induced IP-10 for LTBI diagnosis in high-risk groups were included. A bivariate random-effects model and a hierarchical summary receiver operating characteristic model were used to estimate and summarize the overall diagnostic performance.

**Results:**

Nineteen articles comprising 24 studies (2,014 participants) were included. The pooled sensitivity, specificity, positive and negative likelihood ratios, diagnostic odds ratio, and area under the curve of Mtb antigen-induced IP-10 were 0.81, 0.88, 6.93, 0.21, 32.56, and 0.91, respectively, indicating good diagnostic performance of Mtb antigen-induced IP-10 for detecting LTBI among high-risk groups. The bivariate boxplot, meta-regression and subgroup analysis identified no significant source of heterogeneity.

**Conclusion:**

Mtb antigen-induced IP-10 is a promising biomarker for detecting LTBI among high-risk groups. It may serve as a useful adjunct to IGRAs and TST, although its clinical utility requires further validation.

**Systematic review registration:**

https://www.crd.york.ac.uk/prospero/, identifier CRD420261388089.

## Introduction

Tuberculosis (TB) remains a leading cause of death from a single infectious agent worldwide (1). The World Health Organization (WHO) Global Tuberculosis Report 2025 estimated 10.7 million new cases and 1.23 million deaths in 2024, underscoring a persistent burden despite therapeutic advances (2, 3). Latent tuberculosis infection (LTBI), defined as a state of persistent immune response to *Mycobacterium tuberculosis* (Mtb) without clinical or radiological evidence of active TB (ATB), is estimated to affect nearly one-quarter of the world’s population and constitutes a substantial reservoir for future TB transmission (4, 5). Although only 5-10% of infected individuals will progress to ATB during their lifetime, this risk is markedly elevated in high-risk groups, including contacts of TB cases, healthcare workers, prisoners, children, pregnant women, immigrants from high TB burden regions, individuals with immunocompromising conditions, and patients with silicosis, diabetes mellitus (DM), etc (6–8). Notably, DM increases the risk of progression from LTBI to ATB, partly through impaired protective Th1-type immune responses (9). Thus, accurate LTBI detection, particularly in these high-risk groups, remains a cornerstone of the WHO End TB Strategy.

The diagnosis of LTBI remains challenging because no universally accepted reference standard exists. Currently, the tuberculin skin test (TST) and interferon-γ release assays (IGRAs) are the principal methods used for LTBI screening, particularly in high-risk groups (10, 11). Although the TST is inexpensive and widely available, its performance may be affected by prior Bacillus Calmette-Guérin (BCG) vaccination, exposure to non-tuberculous mycobacteria (NTM), malnutrition, and immune dysfunction (12). IGRAs, including QuantiFERON-TB Gold In-Tube (QFT-GIT), QuantiFERON-TB Gold Plus (QFT-Plus), and T-SPOT.TB, demonstrate superior specificity in BCG-vaccinated individuals, those exposed to NTM, and selected immunocompromised populations (6, 13, 14). Nevertheless, indeterminate results remain a significant challenge, occurring in approximately one in every 26 LTBI screening tests (14, 15). The burden of indeterminate results is particularly pronounced in children, especially those younger than 5 years of age (16). Collectively, these limitations underscore the need for novel biomarkers and improved diagnostic strategies to enhance LTBI detection and risk stratification, particularly among populations at heightened risk of progression to ATB.

Interferon-γ-inducible protein 10 (IP-10), a chemokine, is markedly upregulated following Mtb infection, with expression levels reported to be approximately 100-fold higher than those of IFN-γ. Moreover, IP-10 responses appear to be relatively unaffected by age, sex, TB site, or clinical presentation (17). Notably, among individuals living with HIV and other immunocompromised populations, Mtb antigen-induced IP-10 responses are less affected by reduced CD4^+^ T-cell counts than interferon-γ (IFN-γ), suggesting that IP-10 may serve as a complementary biomarker for LTBI detection in these vulnerable populations (18). Recent evidence suggests that Mtb antigen-induced IP-10 may provide additional diagnostic value for LTBI screening among high-risk populations (9, 19–36). Nevertheless, reported findings on its diagnostic performance remain inconsistent (9, 19–36). Therefore, we conducted a systematic review and meta-analysis to comprehensively evaluate the overall accuracy of Mtb antigen-induced IP-10 for the detection of LTBI in high-risk groups.

## Materials and methods

### Search strategy

A comprehensive literature search was performed in accordance with the Preferred Reporting Items for Systematic Reviews and Meta-Analyses of Diagnostic Test Accuracy Studies (PRISMA-DTA) 2018 and 2020 statements (37, 38). The study protocol was prospectively registered in the International Prospective Register of Systematic Reviews (PROSPERO; CRD420261388089). Five electronic databases, including PubMed, Web of Science, Embase, Scopus, and the Cochrane Library, were systematically searched from database inception through April 2026, with no restrictions on publication year. Only English-language publications were included. The search strategy combined controlled vocabulary terms and free-text keywords related to LTBI and IP-10, including latent tuberculosis infection, LTBI, interferon-γ-inducible protein 10, IP-10, and CXCL10. The full database-specific search strategies are provided in the Supplementary Material. To maximize retrieval, the reference lists of all eligible articles and relevant review papers were manually screened for additional studies. As this study was based exclusively on published data, institutional ethics approval was not required.

### Selection of studies

Two reviewers (LH and QM) independently screened all retrieved records by examining titles, abstracts, and full-text articles. Any disagreements were resolved through consensus discussion. Studies were included if they met predefined eligibility criteria structured according to the PICOS framework. Eligible populations comprised individuals with LTBI and non-TB controls from potential high-risk populations, including close contacts of TB cases, healthcare workers, children, pregnant women, patients with chronic kidney disease (CKD), immune-related disorders (IRD), rheumatoid arthritis (RA), DM and immigrants from high TB burden regions, etc (6–8). The index test was Mtb antigen-induced IP-10 measured in peripheral blood. The reference standard for LTBI diagnosis was based on IGRAs, including QFT-GIT, QFT-Plus, and T-SPOT.TB, TST, or a combination of both. Studies were required to provide sufficient data to calculate or directly extract sensitivity and specificity of IP-10 for LTBI detection in high-risk populations. Eligible study designs included randomized controlled trials, cohort studies (prospective or retrospective), and cross-sectional studies, whereas case-control studies were excluded to minimize the risk of spectrum bias. When multiple diagnostic thresholds of IP-10 were reported within a single study, the optimal cut-off identified by the original study authors was used for analysis. Excluded studies comprised reviews, systematic reviews, case reports, case series, preclinical experiments, and guidelines.

### Data extraction and management

Two reviewers (LH and QM) independently extracted data from all eligible studies using a standardized extraction form. Any inconsistencies were resolved through discussion until consensus was reached. Extracted information included first author, publication year, country, recruiting center, study design, TB high-risk groups, number of participants, and age distribution (including LTBI cases and non-TB controls). When available, risk-related variables were also collected, such as BCG vaccination status and HIV co-infection status. Additional data were extracted on background TB burden, TB/HIV co-infection burden status, reference standard for LTBI diagnosis, IP-10 assay protocols, IP-10 diagnostic cut-off values, and reported sensitivity and specificity. For quantitative synthesis, numbers of true positives (TP), false positives (FP), false negatives (FN), and true negatives (TN) were extracted whenever available.

### Methodological quality assessment

The methodological quality of the included studies was evaluated using the Quality Assessment of Diagnostic Accuracy Studies 2 (QUADAS-2) tool (39). Two reviewers (LH and QM) independently conducted the assessment, and any disagreements were resolved by consensus discussion. QUADAS-2 was used to appraise the risk of bias and applicability concerns for each included study. Summary figures of the quality assessment were produced using Review Manager software (RevMan, version 5.4; Cochrane, London, UK).

### Statistical analysis

All statistical analyses were performed using Stata software (version 19.0; StataCorp, College Station, TX, USA). Pooled estimates of sensitivity, specificity, positive likelihood ratio (PLR), negative likelihood ratio (NLR), and diagnostic odds ratio (DOR) were derived using a bivariate random-effects model, which accounts for both within- and between-study variability in diagnostic accuracy measures. Overall diagnostic performance was assessed by the summary receiver operating characteristic (SROC) curve and the area under the curve (AUC), where higher values indicate better discriminatory ability of Mtb antigen-induced IP-10 for detecting LTBI among high-risk groups (40, 41). In general, an AUC between 0.75 and 0.93 was considered to indicate good diagnostic performance, whereas values exceeding 0.93 suggested excellent accuracy (42). A hierarchical summary receiver operating characteristic (HSROC) model was additionally constructed to provide a global summary of test performance (43).

Statistical heterogeneity was assessed using the I² statistic, with values greater than 75% indicating substantial heterogeneity (44). Bivariate boxplots were constructed to visualize potential outliers and between-study heterogeneity using the joint distribution of sensitivity and specificity (45). When substantial heterogeneity was detected, meta-regression and subgroup analysis were performed to explore potential sources of heterogeneity. Examined subgroup variables included study design (cohort or cross-sectional), TB high-burden (yes or no), TB/HIV high-burden (yes or no), age group (adults or not), method of IP-10 (ELISA or not), blinding of IP-10 testing (yes or unclear) and reference standard (IGRA only or not). Publication bias was evaluated using Deeks’ funnel plot asymmetry test, with p < 0.10 indicating potential publication bias (46).

## Results

### Search results

A total of 998 records were identified from electronic database searches. After removing 565 duplicates, 433 records were screened based on titles and abstracts. At this stage, 384 records were excluded due to irrelevance, including studies involving non-target diseases or populations (n = 162), absence of IP-10 testing (n = 92), irrelevant diagnostic accuracy outcomes (n = 37), reviews, case reports or guidelines (n = 56), and preclinical studies (n = 37). Subsequently, 49 full-text articles were assessed for eligibility, among which 30 articles were excluded because they focused on TB rather than LTBI, lacked sufficient data for 2 × 2 table construction, involved combined biomarkers, reported unstimulated IP-10, or contained overlapping datasets. Ultimately, 19 articles were included in the final meta-analysis (9, 19–36), as shown in **Figure 1**.

**Figure 1:**
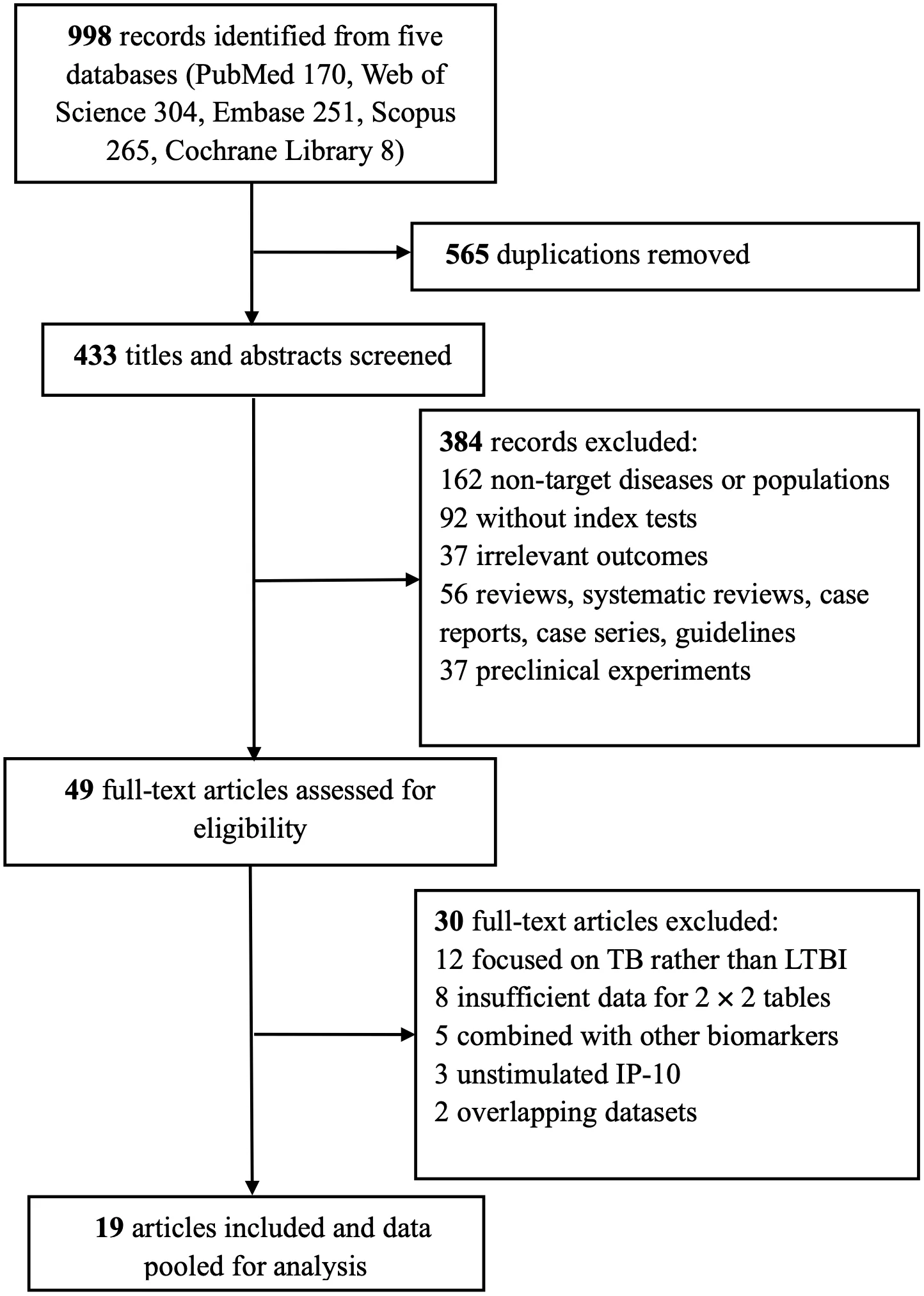
PRISMA flow chart of the process of the search strategy for study selection.

### Characteristics of the included articles

A total of 19 articles comprising 24 studies were included in this meta-analysis (**Table 1**). An article could contribute two studies when IP-10 was evaluated under two different Mtb antigen-stimulation conditions (19, 24, 25, 34, 35). Overall, 2,014 participants were analyzed, including 1,078 individuals with LTBI and 936 non-TB controls. Across the included studies, a total of 2,744 samples were analyzed. The number of samples exceeded the number of participants because five articles analyzed two samples from the same participant for IP-10 measurement following different Mtb antigen-stimulation conditions (19, 24, 25, 34, 35). The studies were conducted across Asia, Africa, Europe, and South America between 2011 and 2025, and were predominantly hospital-based cross-sectional or cohort designs. LTBI populations consisted of high-risk groups, including close contacts of TB patients, children (21, 25, 26, 36), healthcare workers (30, 32), and patients with comorbid conditions, such as CKD (20), DM (9), IRD (22), and RA (35). Participants ranged from children to older adults. BCG vaccination status was inconsistently reported across studies, although most cohorts included both vaccinated and unvaccinated individuals, reflecting regional variability. Only two articles reported that some participants were co-infected with HIV (31, 36). Blinding of index test interpretation was generally unclear, with only four articles explicitly reporting blinded assessment (26, 28, 29, 34).

**Table 1:** Characteristics of the included studies.

Author	Year	Country	Recruiting center	Study design	TB high-risk groups	Participants (N)		Age (years)		BCG-vaccinated (N)		HIV-coinfected (N)	Index test blind?
						LTBI	Non-TB	LTBI	Non-TB	LTBI	Non-TB		
De la Pena Galindo N (19)	2025	Peru	Hospital and health center	Cross-sectional	Contacts of TB patients	17	28	≥22	≥22	16	26	Un	Un
da Silva JCB (20)	2025	Brazil	Medical center	Cross-sectional	CKD and hemodialysis patients	39	80	≥18	≥18	28	76	Un	Un
Ssekamatte P (9)	2025	Uganda	Hospital	Cross-sectional	DM patients	51	12	47 (40-54)	43 (32-55)	44	10	0	Un
Strzelak A (21)	2024	Poland	Hospital and treatment center	Cross-sectional	Children contacts of PTB patients	48	41	<18	<18	48	40	0	Un
Ortakoylu MG (22)	2022	Turkey	Pulmonology clinic	Cross-sectional	IRD patients	21	49	47 ± 14		15	39	0	Un
Indrati AR (23)	2022	Indonesia	Hospital	Cross-sectional	Pregnant women exposed to ATB patients	45	78	29 ± 6	27 ± 5	Un	Un	Un	Un
Ghanaiee RM (24)	2022	Iran	Regional TB reference center	Cohort	Household contacts of PTB patients	285	136	≥18	≥18	Un	Un	Un	Un
Ghanaie RM (25)	2021	Iran	Regional TB reference center	Cohort	Pediatric household contacts of TB patients	104	123	8.5 (1-17)		104	123	Un	Un
El-Sheikh N (26)	2021	Egypt	Hospital	Cross-sectional	Pediatric household contacts of ATB patients	33	21	7 (0.5-17)	10 (0.5-17)	33	21	Un	Yes
Wang S (27)	2018	China	Hospital	Cohort	Household contacts of ATB patients	34	26	43 (15-62)	39 (21-58)	25	23	0	Un
Balcells ME (28)	2018	Chile	Hospital	Cohort	Household contacts of ATB patients	39	33	38 (15-73)	35 (15-76)	34	31	0	Yes
La Manna MP (29)	2018	Italy	Hospital	Cohort	Household or equivalent close contacts of PTB patients	32	20	17-84	24-76	Un	Un	0	Yes
Nonghanphithak D (30)	2017	Thailand	Hospital	Cohort	Close contacts of ATB patients or healthcare workers	38	39	45 ± 12	40 ± 14	26	39	0	Un
Biraro IA (31)	2016	Uganda	Health center	Cohort	Household contacts of TB patients	62	66	0-30	0-30	41	55	8	Un
Rubbo PA (32)	2012	France	Hospital	Cohort	Healthcare workers	8	17	44 (36-50)		8	17	0	Un
Wang S (33)	2012	China	Hospital	Cross-sectional	Household contacts of TB patient	27	45	41 (18-83)		54		0	Un
Borgstrom E (34)	2012	Sweden	Hospital	Cohort	TB-exposed individuals or residents of high-TB-burden areas	23	8	44 (20-67)	47 (23-76)	18	5	0	Yes
Chen DY (35)	2011	Taiwan	Hospital	Cohort	RA patients	24	28	>18	>18	22	26	Un	Un
Yassin MA (36)	2011	Ethiopia	Hospital and health center	Cross-sectional	Children contacts of PTB patients	148	86	8 (1-15)		some		some	Un

LTBI, latent tuberculosis infection; TB, tuberculosis; ATB, active tuberculosis; PTB, pulmonary tuberculosis; CKD, chronic kidney disease; DM, diabetes mellitus; IRD, inflammatory rheumatic diseases; RA, rheumatoid arthritis; BCG, Bacillus Calmette–Guérin; HIV, human immunodeficiency virus.

IP-10 was measured using ELISA, multiplex immunoassay, or RT-PCR, with diagnostic cut-off values varying across studies and Mtb antigen-stimulation conditions. In addition, TB burden, TB/HIV co-infection burden status, reference standards for LTBI diagnosis, and diagnostic performance estimates of Mtb antigen-induced IP-10 for detecting LTBI in high-risk populations are summarized in **Supplementary Table 1**.

### Methodological quality of the eligible articles

The QUADAS-2 framework was used to appraise methodological quality, revealing an overall low risk of bias (**Figure 2**). Regarding patient selection, only one article (5%) was categorized as having an unclear risk of bias, as it lacked details on consecutive enrollment and the timing of LTBI patient inclusion (30). Concerning index test, 11 articles (58%) were judged to have an unclear risk of bias because blinding of IP-10 assessment and pre-specification of the IP-10 threshold were not clearly reported. The high proportion of unclear risk in this domain reflects insufficient methodological reporting and may limit confidence in the assessment of index-test bias. For reference standard, two articles were rated as having an unclear risk of bias because of indeterminate QFT-Plus or QFT-GIT results (20, 33). For flow and timing, six articles (32%) had an unclear risk of bias, as data were missing for LTBI patients or non-TB controls. Applicability concerns for these eligible articles were relatively low.

**Figure 2:**
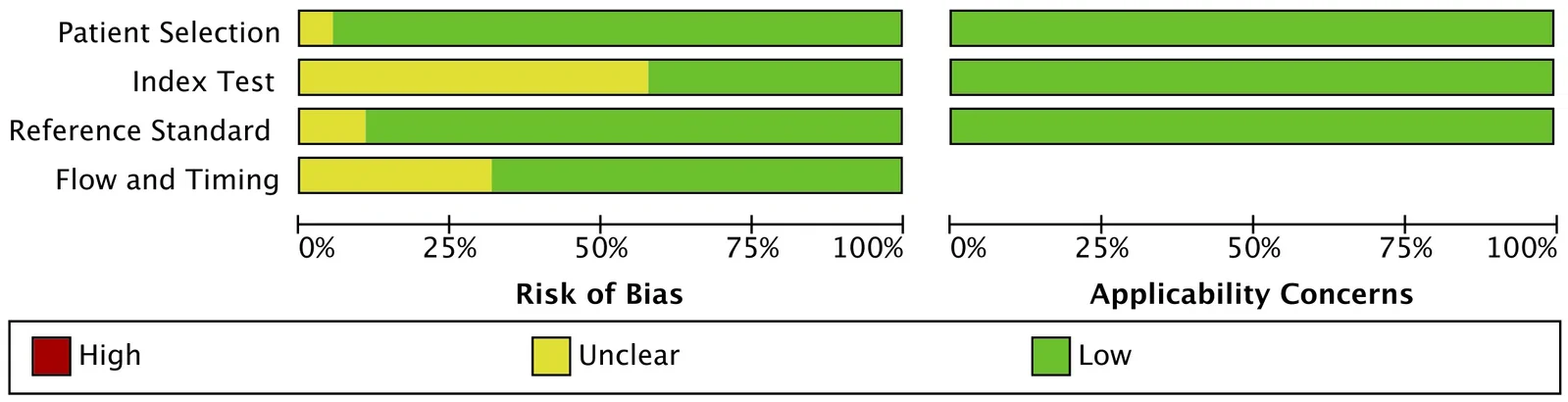
Methodological quality.

### Diagnostic accuracy of Mtb antigen-induced IP-10

We analyzed 24 studies encompassing 2,744 samples derived from 2,014 participants to evaluate the diagnostic accuracy of Mtb antigen-induced IP-10 for detecting LTBI among high-risk groups. The sensitivity varied from 0.54 to 1.00, with a pooled estimate of 0.81 (95% CI: 0.76-0.86; I² = 76%). The specificity ranged from 0.61 to 1.00, yielding a pooled estimate of 0.88 (95% CI: 0.83-0.92; I² = 80%). The pooled PLR and NLR were 6.93 (95% CI: 4.71-10.21; I² = 69%) and 0.21 (95% CI: 0.16-0.28; I² = 78%), respectively. The pooled DOR was 32.56 (95% CI: 19.12-55.43), supporting good discriminatory ability of IP-10 in differentiating LTBI patients from non-TB controls. The SROC curve showed good overall diagnostic performance of IP-10 for LTBI detection, with an AUC of 0.91 (95% CI: 0.88-0.93) (**Supplementary Figure 1**). Likewise, the HSROC curve showed that the summary operating point was located near the upper-left region, further indicating good overall diagnostic performance of IP-10 (**Figure 3**).

**Figure 3:**
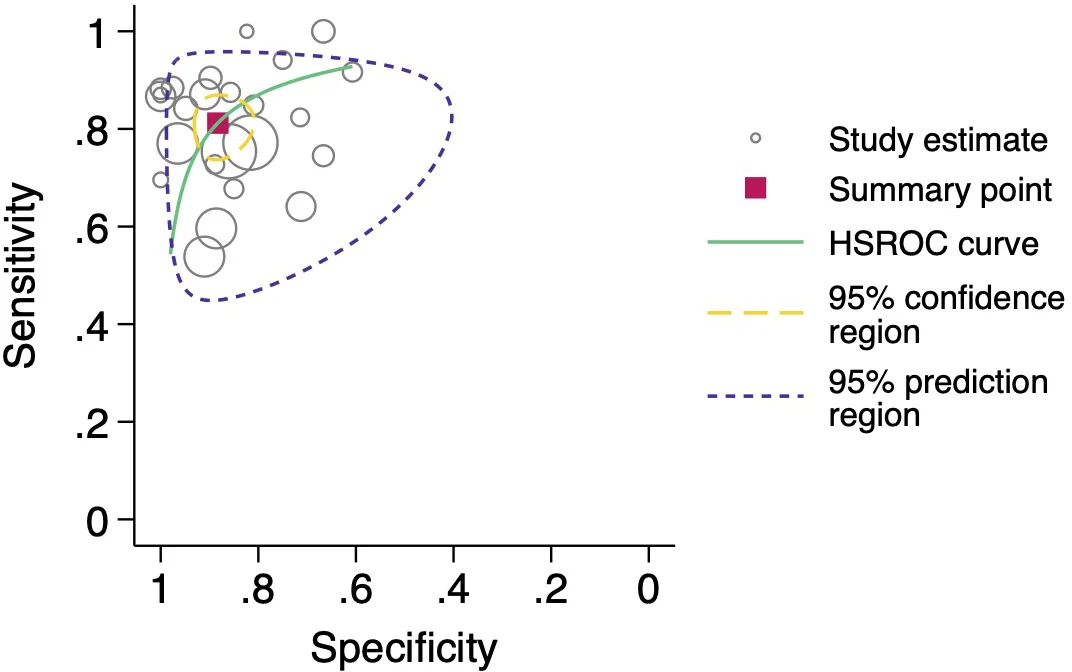
The hierarchical summary receiver operating characteristic curve for assessment of Mtb antigen-induced IP-10 for LTBI among high-risk groups.

### Heterogeneity

Substantial heterogeneity was noted across the included studies, with I² values exceeding 75%. The bivariate boxplot identified two potential outlier studies (**Supplementary Figure 2**) (21, 33). To evaluate their impact, we performed a sensitivity analysis by removing these two studies sequentially. After each exclusion, the pooled sensitivity varied between 0.80 and 0.81, specificity between 0.87 and 0.89, and the AUC between 0.90 and 0.91. These results suggest that the outliers did not substantially influence the overall diagnostic accuracy of Mtb antigen-induced IP-10 for LTBI among high-risk groups. Moreover, these outlying studies were not the primary source of the heterogeneity.

Meta-regression and subgroup analysis further revealed no statistically significant differences according to study design (cohort or cross-sectional), TB high-burden (yes or no), TB/HIV high-burden (yes or no), age group (adults or not), method of IP-10 (ELISA or not), IP-10 test blind (yes or unclear) and reference standard (IGRA only or not), with corresponding p values of 0.36, 0.41, 0.41, 0.09, 0.85, 0.66, and 0.21, respectively. These findings suggest that these variables were probably not the principal drivers of the heterogeneity. The pooled estimates of sensitivity and specificity for each subgroup are summarized in **Table 2**.

**Table 2:** Heterogeneity assessment for IP-10 detecting LTBI among high-risk groups.

Covariates		Studies (N)	Samples (N)	Sensitivity (95%)	Specificity (95%)	P value
Study design	Cohort	14	1852	0.78(0.72-0.85)	0.89(0.84-0.95)	0.36
	Cross-sectional	10	892	0.85(0.78-0.91)	0.87(0.79-0.94)	
TB high-burden	Yes	8	876	0.84(0.77-0.91)	0.91(0.85-0.97)	0.41
	No	16	1868	0.80(0.73-0.86)	0.87(0.81-0.92)	
TB/HIV high-burden	Yes	8	876	0.84(0.77-0.91)	0.91(0.85-0.97)	0.41
	No	16	1868	0.80(0.73-0.86)	0.87(0.81-0.92)	
Age group	Adults	17	1758	0.83(0.78-0.88)	0.86(0.80-0.91)	0.09
	Children/children and adults	7	986	0.75(0.66-0.84)	0.92(0.87-0.97)	
Method of IP-10	ELISA	14	2290	0.81(0.74-0.87)	0.89(0.84-0.94)	0.85
	Multiplex immunoassay/RT-PCR	10	454	0.82(0.75-0.90)	0.87(0.78-0.95)	
IP-10 blind	Yes	5	216	0.77(0.66-0.89)	0.91(0.82-1.00)	0.66
	Un	19	2528	0.82(0.77-0.87)	0.88(0.83-0.93)	
Reference standard	QFT	11	748	0.85(0.79-0.91)	0.87(0.79-0.94)	0.21
	TST/TST and/or QFT	13	1996	0.78(0.71-0.84)	0.89(0.84-0.95)	

IP-10, interferon γ-induced protein 10; LTBI, latent tuberculosis infection; TB, tuberculosis; HIV, human immunodeficiency virus; ELISA, enzyme-linked immunosorbent assay; RT-PCR, reverse transcription polymerase chain reaction; QFT, QuantiFERON-TB; TST, tuberculin skin test.

### Publication bias

No significant publication bias was detected by Deeks’ test (p = 0.19) when distinguishing LTBI patients from non-TB controls (**Supplementary Figure 3**).

## Discussion

Among close contacts of TB patients, the prevalence of LTBI is estimated to range from 30% to 50%, and the likelihood of progression to ATB is greatest during the first two years following infection (6). In children, LTBI affects approximately 20%–30% of the population, with those younger than five years facing the highest risk of developing ATB (6). The performances of current immunological assays for LTBI detection are influenced by age, BCG vaccination status, and host immune function (13). These limitations underscore the need for complementary biomarkers that can improve the identification of LTBI, particularly in populations at increased risk of disease progression.

In the present meta-analysis, we synthesized evidence from 19 original articles comprising 24 studies to determine the diagnostic value of Mtb antigen-induced IP-10 for identifying LTBI among high-risk groups. The pooled estimates demonstrated a sensitivity of 0.81, a specificity of 0.88, and an AUC of 0.91, indicating good overall diagnostic performance. Although two studies were identified as potential outliers on the bivariate boxplot, further analyses indicated that they were not the principal contributors to the observed heterogeneity. None of the prespecified variables—including study design, TB burden, TB/HIV burden, age group, IP-10 assay method, blinding of IP-10 testing, and reference standard—significantly influenced heterogeneity in meta-regression or subgroup analysis.

In 2019, a meta-analysis reported that IP-10 achieved a sensitivity of 0.86 and a specificity of 0.88 for the diagnosis of pulmonary TB (47), while a 2024 systematic review found that IP-10 distinguishes ATB from LTBI with a pooled sensitivity of 0.72 and a specificity of 0.78 (48). In 2019, our group published a meta-analysis on IP-10 for LTBI detection, synthesizing 12 articles across 15 studies with a total of 1,122 participants (13). It yielded a pooled sensitivity of 0.85 and specificity of 0.89, supporting the potential diagnostic performance of IP-10 (13). However, that meta-analysis included studies involving both healthy individuals and high-risk populations, potentially introducing spectrum bias. Moreover, several included studies employed a case-control design, which is known to overestimate diagnostic accuracy in real-world settings (49). Nevertheless, the overall diagnostic accuracy of Mtb antigen-induced IP-10 for LTBI among high-risk groups has not been comprehensively established. In the present study, only cohort and cross-sectional studies were included to better reflect routine clinical practice. The pooled sensitivity and specificity of Mtb antigen-induced IP-10 were 0.81 and 0.88, corresponding to false-negative and false-positive rates of 19% and 12%, respectively. The AUC of 0.91 further indicates good discriminatory ability for identifying LTBI among high-risk individuals. Clinically, rather than replacing existing tests, we propose that IP-10 be used alongside IGRAs and TST, where it may enhance the identification of LTBI in populations at increased risk of progression to ATB.

Age-related differences in anti-Mtb immunity between children and adults represent a potential source of between-study variability (50); however, this factor did not significantly influence heterogeneity in the current analysis. ELISA, multiplex immunoassays, and RT-PCR exhibited comparable diagnostic performance and. Subgroup analysis showed that ELISA achieved sensitivity and specificity similar to those of multiplex immunoassays or RT-PCR (0.81 vs. 0.82 and 0.89 vs. 0.87, respectively). Considering its comparable performance, lower cost, and broader availability, ELISA may represent a practical and accessible option for IP-10 detection in routine practice.

### Strengths and limitations

This study has several strengths. It focused specifically on high-risk populations and included only cohort and cross-sectional studies, thereby providing a comprehensive assessment of the diagnostic performance of Mtb antigen-induced IP-10 in these populations.

Several limitations of this study warrant consideration. First, because IP-10 is frequently quantified in combination with other immunological markers, the concurrent measurement of additional analytes may attenuate the apparent diagnostic performance of IP-10 as a standalone indicator, thereby complicating the assessment of its independent reliability and added clinical value. Second, the use of study-specific optimal IP-10 cut-offs reported by the original studies may have introduced threshold-selection bias and potentially overestimated the pooled diagnostic performance. Third, although IGRA and TST are commonly used as reference standards for LTBI in the absence of a definitive gold standard, their limitations may lead to potential misclassification and affect the pooled diagnostic accuracy estimates of IP-10. Fourth, substantial heterogeneity was observed in sensitivity, specificity, and NLR. Although the examined sources of heterogeneity—including study design, TB burden, TB/HIV burden, age group, IP-10 assay method, blinding of IP-10 testing, and reference standard—did not emerge as significant contributors, the sources of heterogeneity remain uncertain. Further exploration according to risk of bias for QUADAS-2 was limited by the absence of studies judged to be at high risk. The variability in IP-10 cut-off thresholds across studies may have contributed to the observed between-study inconsistency; however, the wide dispersion of reported cut-offs precluded a meaningful bivariate meta-regression stratified by threshold values. Fifth, clinical heterogeneity should also be considered, as the included high-risk populations differed in age, underlying conditions, and TB burden settings, which may have contributed to variability in IP-10 responses and diagnostic performance across studies. Finally, while Deeks’ funnel plot did not reveal significant asymmetry, a non-significant result does not exclude the possibility of publication bias. Our search was restricted to English-language publications, and we did not formally evaluate the potential impact of studies published in other languages. Thus, the possibility of selective reporting cannot be entirely excluded.

In conclusion, Mtb antigen-induced IP-10 is a promising biomarker for detecting LTBI among high-risk groups. Clinically, rather than replacing existing tests, IP-10 may have potential for use alongside IGRAs and TST and may provide additional diagnostic information for the identification of LTBI in populations at increased risk of progression to ATB. Further prospective studies using standardized assay protocols and diagnostic thresholds are warranted to confirm its clinical utility.

## Data Availability

The original contributions presented in the study are included in the article/**Supplementary Material**. Further inquiries can be directed to the corresponding author.
