# Supplementary material for: Diagnostic accuracy of Mycobacterium tuberculosis antigen-induced interferon-γ-inducible protein 10 for latent tuberculosis infection among high-risk groups: a systematic review and meta-analysis
Source: Front Immunol. 2026 Sep 28;17:1951983. doi: 10.3389/fimmu.2026.1951983 (PMC13654728; doi:10.3389/fimmu.2026.1951983)
Supplement: Supplementary file 1 [file DataSheet1.pdf]

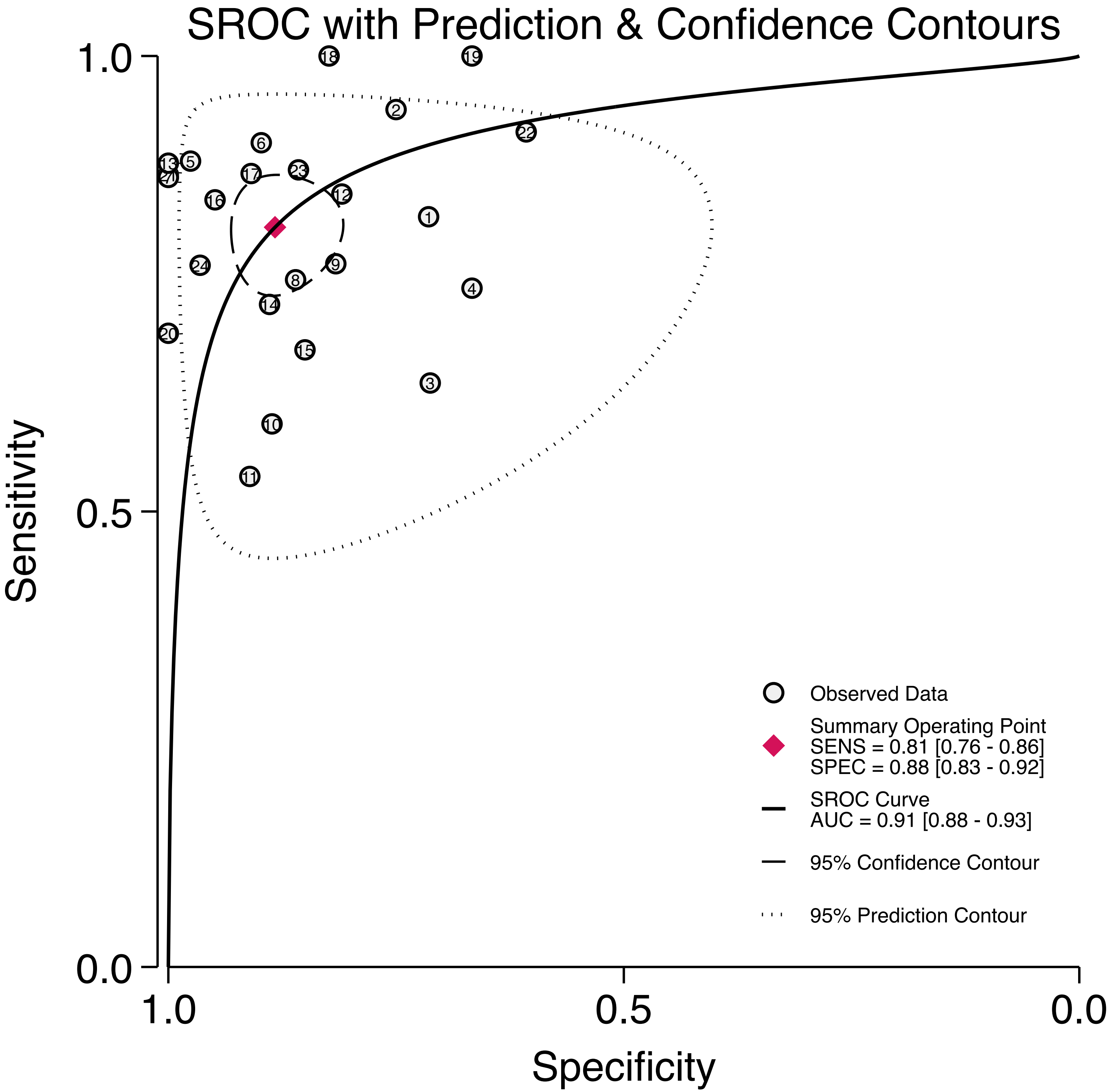

SROC with Prediction & Confidence Contours
1.0
0.5
0.0
Sensitivity
1.0
0.5
0.0
Specificity
Observed Data
Summary Operating Point
SENS = 0.81 [0.76 - 0.86]
SPEC = 0.88 [0.83 - 0.92]
SROC Curve
AUC = 0.91 [0.88 - 0.93]
95% Confidence Contour
95% Prediction Contour
