# Supplementary material for: Diagnostic accuracy of Mycobacterium tuberculosis antigen-induced interferon-γ-inducible protein 10 for latent tuberculosis infection among high-risk groups: a systematic review and meta-analysis
Source: Front Immunol. 2026 Sep 28;17:1951983. doi: 10.3389/fimmu.2026.1951983 (PMC13654728; doi:10.3389/fimmu.2026.1951983)
Supplement: Supplementary file 2 [file DataSheet2.pdf]

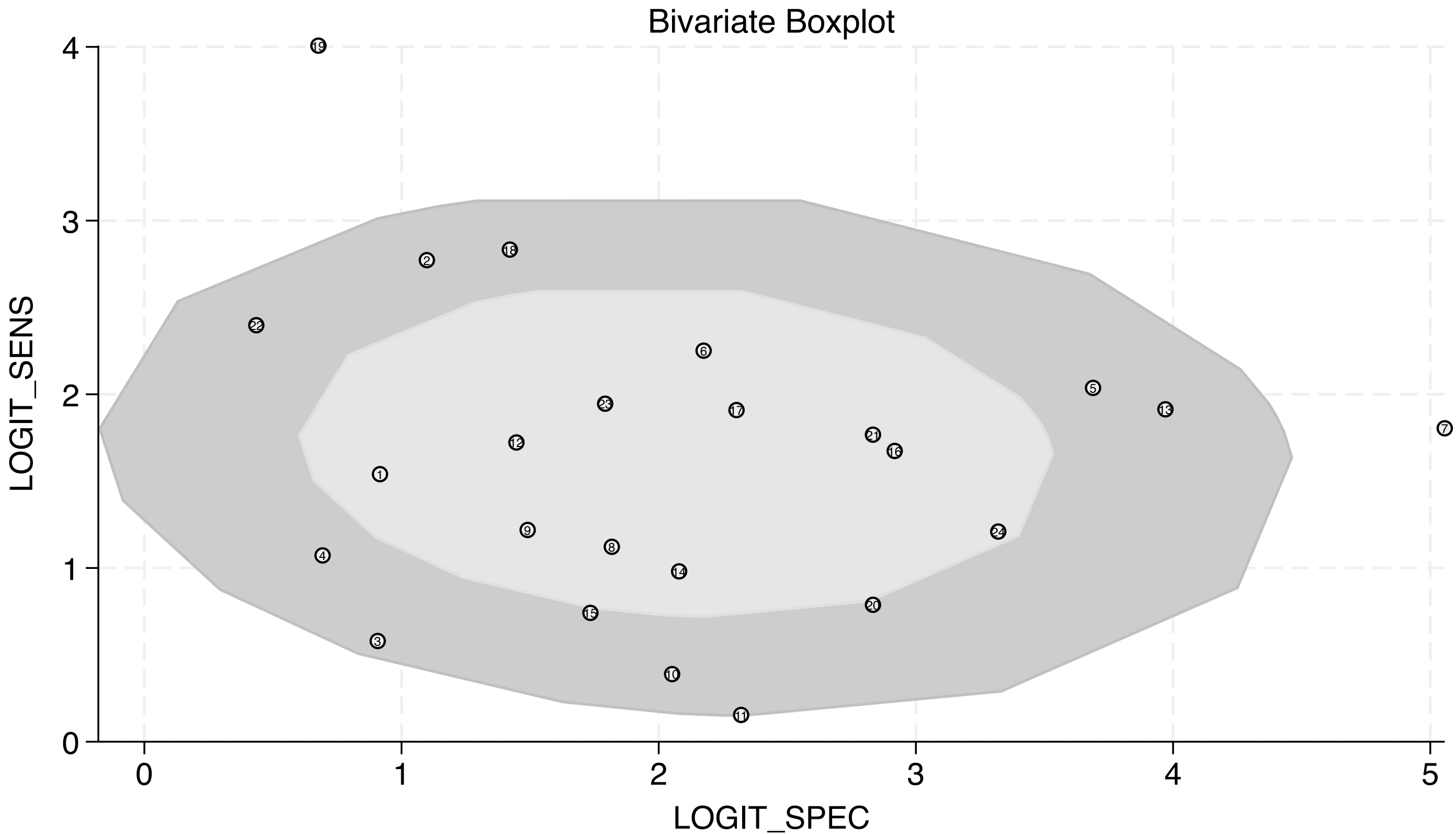
Bivariate Boxplot
LOGIT_SENS
LOGIT_SPEC
0
1
2
3
4
5
19
18
2
22
6
23
17
5
13
7
21
16
12
1
9
8
24
4
14
20
15
3
10
11
