# Supplementary material for: Diagnostic accuracy of Mycobacterium tuberculosis antigen-induced interferon-γ-inducible protein 10 for latent tuberculosis infection among high-risk groups: a systematic review and meta-analysis
Source: Front Immunol. 2026 Sep 28;17:1951983. doi: 10.3389/fimmu.2026.1951983 (PMC13654728; doi:10.3389/fimmu.2026.1951983)
Supplement: Supplementary file 3 [file DataSheet3.pdf]

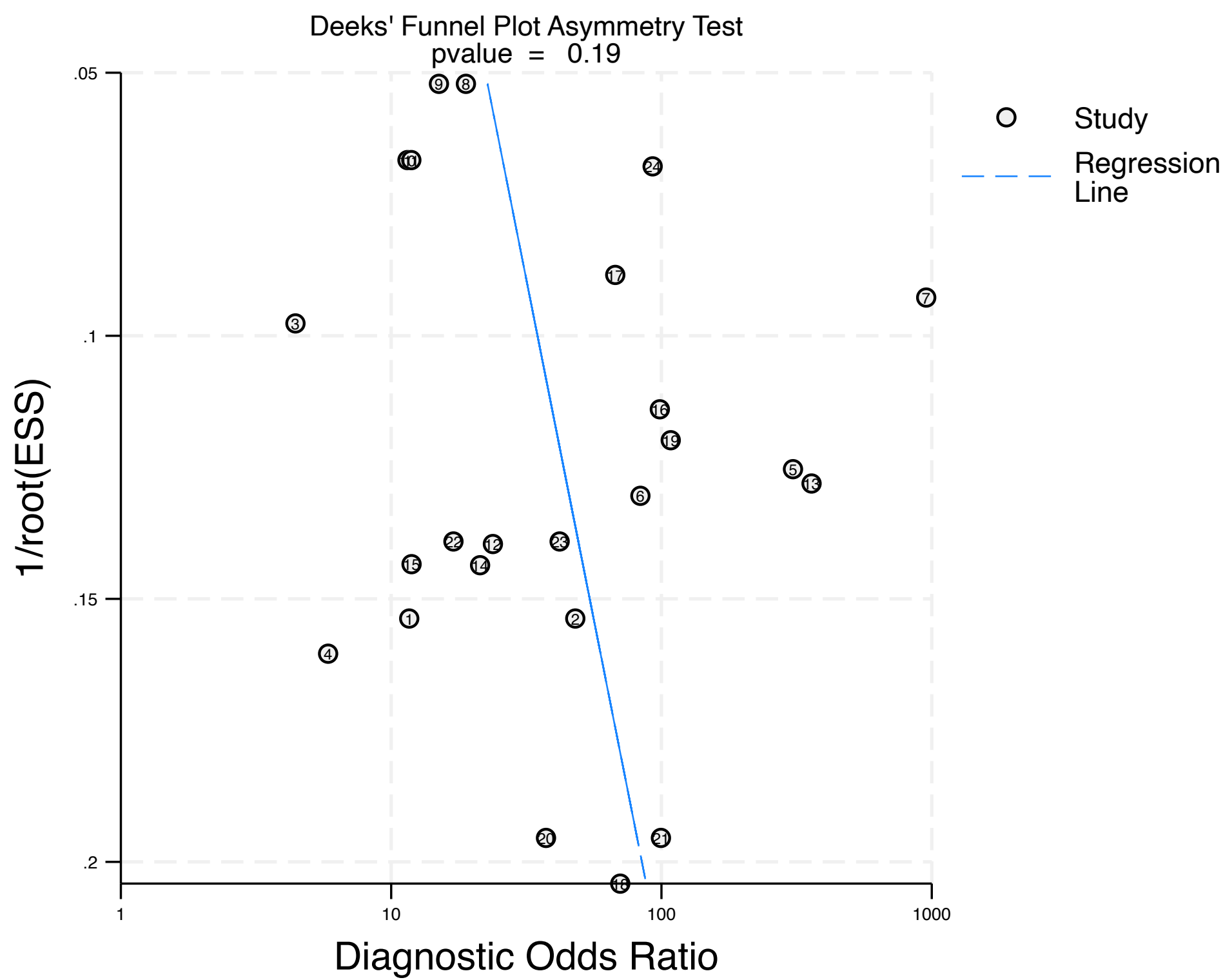

Deeks' Funnel Plot Asymmetry Test
pvalue = 0.19
1/root(ESS)
.05
.1
.15
.2
Diagnostic Odds Ratio
1
10
100
1000
Study
Regression Line
