# Supplementary material for: Diagnostic accuracy of Mycobacterium tuberculosis antigen-induced interferon-γ-inducible protein 10 for latent tuberculosis infection among high-risk groups: a systematic review and meta-analysis
Source: Front Immunol. 2026 Sep 28;17:1951983. doi: 10.3389/fimmu.2026.1951983 (PMC13654728; doi:10.3389/fimmu.2026.1951983)
Supplement: Supplementary file 4 [file Table1.docx]

**Supplementary table 1. Baseline data and diagnostic performance of the included studies**

| Author | Year | TB high-burden | TB/HIV high-burden | Reference standard of LTBI | Index test (IP-10) | | Sensitivity (%) | Specificity (%) | TP | FP | FN | TN |
| --- | --- | --- | --- | --- | --- | --- | --- | --- | --- | --- | --- | --- |
|  |  |  |  |  | Assay | Cut-off (pg/ml) |  |  |  |  |  |  |
| De la Pena Galindo N | 2025 | No | No | QFT-Plus | Multiplex immunoassay | 485.79 (TB1) | 82 | 71 | 14 | 8 | 3 | 20 |
| De la Pena Galindo N | 2025 | No | No | QFT-Plus | Multiplex immunoassay | 859.3 (TB2) | 94 | 75 | 16 | 7 | 1 | 21 |
| da Silva JCB | 2025 | Yes | Yes | QFT-Plus | ELISA | 535.9 | 64 | 71 | 25 | 23 | 14 | 57 |
| Ssekamatte P | 2025 | Yes | Yes | QFT-Plus | Multiplex immunoassay | 8.66 | 75 | 67 | 38 | 4 | 13 | 8 |
| Strzelak A | 2024 | No | No | TST+QFT-Plus | ELISA | 1498.1 | 88 | 98 | 23 | 1 | 3 | 40 |
| Ortakoylu MG | 2022 | No | No | QFT-GIT | ELISA | 2197 | 90 | 90 | 19 | 5 | 2 | 44 |
| Indrati AR | 2022 | Yes | Yes | QFT-Plus | ELISA | 649.63 | 87 | 100 | 39 | 0 | 6 | 78 |
| Ghanaiee RM | 2022 | No | No | TST and/or QFT-Plus | ELISA | 26.0 (TB1) | 75 | 86 | 215 | 19 | 70 | 117 |
| Ghanaiee RM | 2022 | No | No | TST and/or QFT-Plus | ELISA | 16.15 (TB2) | 77 | 82 | 220 | 25 | 65 | 111 |
| Ghanaie RM | 2021 | No | No | TST and/or QFT-Plus | ELISA | 67.2 (TB1) | 60 | 89 | 62 | 14 | 42 | 109 |
| Ghanaie RM | 2021 | No | No | TST and/or QFT-Plus | ELISA | 142.3 (TB2) | 54 | 91 | 56 | 11 | 48 | 112 |
| El-Sheikh N | 2021 | No | No | TST and/or QFN-GIT | RT-PCR | Un | 85 | 81 | 28 | 4 | 5 | 17 |
| Wang S | 2018 | Yes | Yes | QFT-GIT | Multiplex immunoassay | 2009 | 88 | 100 | 30 | 0 | 4 | 26 |
| Balcells ME | 2018 | No | No | QFT-GIT | Multiplex immunoassay | >42 | 73 | 89 | 16 | 3 | 6 | 24 |
| La Manna MP | 2018 | No | No | TST and QFT-GIT | Multiplex immunoassay | >6226 | 68 | 85 | 21 | 3 | 10 | 17 |
| Nonghanphithak D | 2017 | Yes | Yes | QFT-GIT | ELISA | 6163 | 84 | 95 | 32 | 2 | 6 | 37 |
| Biraro IA | 2016 | Yes | Yes | TST and QFT-GIT | ELISA | 8239 | 87 | 91 | 54 | 6 | 8 | 60 |
| Rubbo PA | 2012 | No | No | QFT-GIT | Multiplex immunoassay | 1259 | 100 | 82 | 8 | 3 | 0 | 14 |
| Wang S | 2012 | Yes | Yes | QFT-GIT | ELISA | 451.3 | 100 | 67 | 27 | 15 | 0 | 30 |
| Borgstrom E | 2012 | No | No | TST | Multiplex immunoassay | 100 (CFP-10) | 70 | 100 | 16 | 0 | 7 | 8 |
| Borgstrom E | 2012 | No | No | TST | Multiplex immunoassay | 72.2 (ESAT-6) | 87 | 100 | 20 | 0 | 3 | 8 |
| Chen DY | 2011 | No | No | TST | ELISA | 586.3 (CFP-10) | 92 | 61 | 22 | 11 | 2 | 17 |
| Chen DY | 2011 | No | No | TST | ELISA | 691.6 (ESAT-6) | 88 | 86 | 21 | 4 | 3 | 24 |
| Yassin MA | 2011 | Yes | Yes | TST and/or QFT-GIT | ELISA | 3022 | 77 | 97 | 114 | 3 | 34 | 83 |

Abbreviations: TB, tuberculosis; LTBI, latent tuberculosis infection; HIV, human immunodeficiency virus; IP-10, interferon-γ-induced protein 10; QFT-Plus, QuantiFERON-TB Gold Plus; QFT-GIT, QuantiFERON-TB Gold In-Tube; TST, tuberculin skin test; ELISA, enzyme-linked immunosorbent assay; RT-PCR, reverse transcription polymerase chain reaction; CFP-10, culture filtrate protein 10; ESAT-6, early secreted antigenic target 6; TP, true positive; FP, false positive; FN, false negative; TN, true negative.
