# Supplementary material for: Diagnostic accuracy of Mycobacterium tuberculosis antigen-induced interferon-γ-inducible protein 10 for latent tuberculosis infection among high-risk groups: a systematic review and meta-analysis
Source: Front Immunol. 2026 Sep 28;17:1951983. doi: 10.3389/fimmu.2026.1951983 (PMC13654728; doi:10.3389/fimmu.2026.1951983)
Supplement: Supplementary file 5 [file Table2.docx]

**Full database-specific search strategies.**

**PubMed**
(("Chemokine CXCL10"[Mesh]) OR (((((((((chemokine CXCL10) OR (interferon gamma-induced protein 10)) OR (interferon γ-induced protein 10)) OR (interferon-inducible protein 10)) OR (small inducible cytokine B10)) OR (gamma IP-10 protein)) OR (cytokine CXCL10)) OR (CXCL10)) OR (IP-10))) AND (("Latent Tuberculosis"[Mesh]) OR (((((latent tuberculosis) OR (latent tuberculoses)) OR (latent tuberculosis infection)) OR (latent tuberculosis infections)) OR (LTBI)))

**Web of Science**
#1 ((((TS=(latent tuberculosis)) OR TS=(latent tuberculoses)) OR TS=(latent tuberculosis infection)) OR TS=(latent tuberculosis infections)) OR TS=(LTBI)

#2 ((((((((TS=(chemokine CXCL10)) OR TS=(interferon gamma-induced protein 10)) OR TS=(interferon γ-induced protein 10)) OR TS=(interferon-inducible protein 10)) OR TS=(small inducible cytokine B10)) OR TS=(gamma IP-10 protein)) OR TS=(cytokine CXCL10)) OR TS=(CXCL10)) OR TS=(IP-10)

#3 #1 AND #2
**Embase**
#1 latent tuberculosis/ or latent tuberculosis.mp.

#2 latent tuberculosis infection.mp.

#3 latent tuberculosis infections.mp.

#4 LTBI.mp.

#5 1 or 2 or 3 or 4

#6 interferon-gamma-induced protein 10.mp. or gamma interferon inducible protein 10/

#7 interferon-inducible protein 10.mp.

#8 chemokine CXCL10.mp.

#9 small inducible cytokine B10.mp.

#10 gamma IP-10 protein.mp.

#11 cytokine CXCL10.mp.

#12 CXCL10.mp.

#13 IP-10.mp.

#14 6 or 7 or 8 or 9 or 10 or 11 or 12 or 13

#15 5 and 14

**Scopus**
( ( TITLE-ABS-KEY ( interferon-gamma-induced protein 10 ) OR TITLE-ABS-KEY ( interferon γ-induced protein 10 ) OR TITLE-ABS-KEY ( chemokine CXCL10 ) OR TITLE-ABS-KEY ( interferon-inducible protein 10 ) OR TITLE-ABS-KEY ( small inducible cytokine B10 ) OR TITLE-ABS-KEY ( gamma IP-10 protein ) OR TITLE-ABS-KEY ( cytokine CXCL10 ) OR TITLE-ABS-KEY ( CXCL10 ) OR TITLE-ABS-KEY ( IP-10 ) ) ) AND ( ( TITLE-ABS-KEY ( latent tuberculosis ) OR TITLE-ABS-KEY ( latent tuberculosis infection ) OR TITLE-ABS-KEY ( latent tuberculosis infections ) OR TITLE-ABS-KEY ( LTBI ) ) )

**Cochrane Library**
#1 MeSH descriptor: [Latent Tuberculosis] explode all trees

#2 (latent tuberculosis):ti,ab,kw OR (latent tuberculosis infection):ti,ab,kw OR (latent tuberculosis infections):ti,ab,kw OR (LTBI):ti,ab,kw

#3 #1 or #2

#4 MeSH descriptor: [Chemokine CXCL10] explode all trees

#5 (chemokine CXCL10):ti,ab,kw OR (interferon-gamma-induced protein 10):ti,ab,kw OR (interferon γ-induced protein 10):ti,ab,kw OR (interferon-inducible protein 10):ti,ab,kw OR (small inducible cytokine B10):ti,ab,kw

#6 (gamma IP-10 protein):ti,ab,kw OR (cytokine CXCL10):ti,ab,kw OR (CXCL10):ti,ab,kw OR (IP-10):ti,ab,kw

#7 #4 or #5 or #6

#8 #3 and #7
